# Genetic variability and ontogeny predict microbiome structure in a disease-challenged montane amphibian

**DOI:** 10.1038/s41396-018-0167-0

**Published:** 2018-06-25

**Authors:** Sarah M. Griffiths, Xavier A. Harrison, Ché Weldon, Michael D. Wood, Abigail Pretorius, Kevin Hopkins, Graeme Fox, Richard F. Preziosi, Rachael E. Antwis

**Affiliations:** 1 School of Science and the Environment, Manchester Metropolitan University, Manchester, UK; 2 Institute of Zoology, Zoological Society of London, London, UK; 3 Unit for Environmental Research and Management, Faculty of Natural Science, North-West University, Potchefstroom, South Africa; 4 School of Environment and Life Sciences, University of Salford, Salford, UK

## Abstract

Amphibian populations worldwide are at risk of extinction from infectious diseases, including chytridiomycosis caused by the fungal pathogen *Batrachochytrium dendrobatidis* (*Bd*). Amphibian cutaneous microbiomes interact with *Bd* and can confer protective benefits to the host. The composition of the microbiome itself is influenced by many environment- and host-related factors. However, little is known about the interacting effects of host population structure, genetic variation and developmental stage on microbiome composition and *Bd* prevalence across multiple sites. Here we explore these questions in *Amietia hymenopus*, a disease-affected frog in southern Africa. We use microsatellite genotyping and 16S amplicon sequencing to show that the microbiome associated with tadpole mouthparts is structured spatially, and is influenced by host genotype and developmental stage. We observed strong genetic structure in host populations based on rivers and geographic distances, but this did not correspond to spatial patterns in microbiome composition. These results indicate that demographic and host genetic factors affect microbiome composition within sites, but different factors are responsible for host population structure and microbiome structure at the between-site level. Our results help to elucidate complex within- and among- population drivers of microbiome structure in amphibian populations. That there is a genetic basis to microbiome composition in amphibians could help to inform amphibian conservation efforts against infectious diseases.

## Introduction

Multicellular organisms act as hosts to a diverse suite of bacterial communities, collectively referred to as the microbiome. Recent research across multiple taxa has highlighted that these bacterial communities perform beneficial functions for the host, including protection from infectious pathogens [1–3]. Despite the importance of symbiotic relationships between microbes and their hosts, a comprehensive understanding of factors that influence the diversity and composition of the microbiome, particularly for non-human animals, is lacking. Though previous studies have revealed landscape-scale variation among populations in microbiome structure [4–6], and fine-scale variation among individuals within populations due to life stage [6], diet [7], gender [7] and genotype [7, 8], few have investigated both within and among population drivers of microbiome structure in a single framework [6, 8]. Studying factors that determine variation in the distribution of bacterial symbionts at both the individual and landscape scale is crucial for understanding how individual susceptibility to pathogens varies within and among populations.

The amphibian skin microbiome represents a model system for understanding the ecological drivers of host microbiome structure and the role of symbiotic bacteria in protecting the host from pathogens. The lethal and globally distributed amphibian chytrid fungus (*Batrachochytrium dendrobatidis; Bd*) is causing mass mortalities and rapid population declines of amphibians around the world and is a major driver of the current amphibian extinction crisis [9]. Certain host-associated bacteria have been shown to influence susceptibility to *Bd*, with particular bacterial genera conferring increased or decreased protection against *Bd* in experimental and field studies [2, 10–12]. Host genotype is one factor that may underpin variation in the presence and persistence of these protective microbes, both within and among host populations [7, 8, 13–16]. For example, polymorphism of the major histocompatibility complex (MHC) Class IIb gene has been shown to co-vary with gut microbiome structure in vertebrates [7]. The expression of anti-microbial peptides (AMPs) on frog skin is governed by innate immunity [17], and so variation in immune genes may drive structural changes in the microbiome through differential AMP expression. The population genetic structure could therefore yield differences in microbiome at the landscape level if it is representative of the non-random distribution of functional genetic variation at immune loci. Furthermore, shifts in the environmental reservoir of bacteria across large geographic scales may also cause differences in the relative abundances of bacteria able to colonise the host [4]. Within populations, fine-scale variation in life-history traits, such as age, may further modify the ability of bacteria to coexist on the skin, or favour differential selection by the host from the environment. Though microbiome differences have been shown among larvae, juveniles and adults in some amphibian species [18, 19], changes in microbiome structure throughout larval ontogeny have not been investigated. This may be especially pertinent, given that fine-scale variation in *Bd* infection loads has been found across developmental stages of tadpoles [20, 21]. Research into the links between host genetics, developmental stage, microbiome composition and pathogen susceptibility may enable a better understanding of the factors governing disease in vulnerable populations.

Here we use a disease-challenged high-altitude amphibian species in the Drakensberg Mountains of southern Africa to investigate within and among population predictors of microbiome structure that may have implications for disease susceptibility. Using microsatellite genotyping and 16S amplicon sequencing, we examine how population genetic structure, individual genotype, and developmental stage determine differences in microbiome structure among individuals. Specifically, we aim to: (i) quantify *Bd* infection across *Amietia hymenopus* frog populations; (ii) characterise the microbiome of *A. hymenopus*; (iii) examine the genetic population structure of *A. hymenopus* on the Drakensberg plateau; (iv) investigate predictors of microbiome structure among sites (specifically river, geographic distance and genetic differentiation among host populations); (v) investigate within-site predictors of microbiome structure (specifically host genotype and ontogenetic stage); and (vi) examine the roles of host microbiome and host genetics in influencing *Bd* infection loads.

## Methods

### Study site

**Fig. 1:**
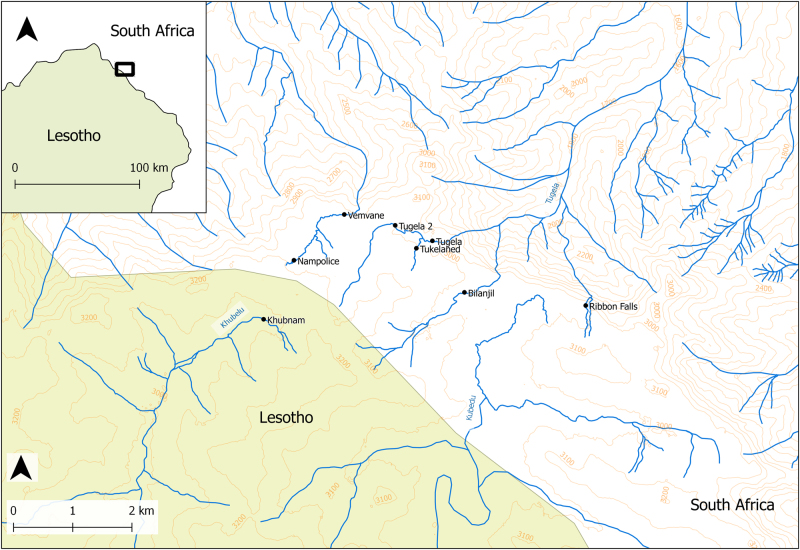
Map of the river system and study sites in Mont-aux-Sources in the Drakensberg Mountains. The rivers originate from springs on the plateau; thus, the rivers in South Africa (Vemvane, Tugela, Bilanjil and Ribbon Falls) all flow south-west to north-east before flowing over the precipice into South Africa; Khubelu in Lesotho flows from north-east to south-west

The Drakensberg Mountains of southern Africa are a vast mountain range reaching up to 3482 m above sea level (asl). Here, the Mont-aux-Sources plateau crosses north-east Lesotho and South Africa. On the South African side, four streams (Vemvane, Tugela, Bilanjil and Ribbon Falls) flow 1–3 km across the plateau from multiple origins in the high peaks on the South Africa-Lesotho border, before dropping off a ~1000 m precipice into South Africa (Fig. 1). Two other streams on the plateau flow into Lesotho; Khubela and Kubedu. The Phofung river frog (*Amietia hymenopus*) is the only amphibian found in most of the Mont-aux-Sources stream system, with the exception of the Kubedu river, where *A. hymenopus* is absent and *A. umbraculata* is present [22]. Long-term (10 year) sampling in this region indicates *A. hymenopus* has a consistent history of *Bd* infection with associated mass mortalities [23].

### Sample collection

We collected tadpoles from Mont-aux-Sources by permission of Ezemvelo KwaZulu Natal Wildlife and the Kingdom of Lesotho Ministry of Tourism, Environment and Culture. We sampled eight sites across five rivers over the plateau; ‘Nampolice’ and ‘Vemvane’ from the Vemvane River (South Africa); ‘Tugela’, ‘Tugela 2’ and ‘Tukelahed’ from the Tugela River (South Africa); ‘Khubnam’ from the Khubela River (Lesotho); and ‘Bilanjil’ and ‘Ribbon Falls’ from their respective rivers (South Africa; Fig. 1). High-resolution mapping was unavailable for the study area, so we used QGIS (version 2.18) to combine Open Source data sets and then digitise the water courses within the study area (Supplementary Information).

We collected 20–28 tadpoles per site (nine at Khubnam) along a ~150 m transect within an altitudinal range of 3010–3090 m asl to minimize variation in *Bd* infection. We conducted two sampling trips 4-weeks apart; in March 2015, we collected tadpoles from six sites (Nampolice, Tugela, Tugela 2, Tukelahed, Bilajil and Ribbon Falls; Fig. 1) and analysed these for *Bd* infection, host genotype and microbiome composition. In April 2015, we collected tadpoles from Vemvane and Khubnam to enhance the population genetics analyses, but did not carry out microbiome analyses on these samples in order to avoid spurious variation caused by temporal changes (Table 1). We stored tadpoles in 95% ethanol prior to Gosner staging [24] and exported carcasses for molecular analyses in the UK by permission of the Department of Agriculture, Forestry and Fisheries, South Africa, and the Department for Environment, Food and Rural Affairs, UK. We excised full mouthparts for DNA extraction as due to their keratinised nature, *Bd* primarily affects this area in tadpoles. DNA was extracted using the DNeasy® Blood and Tissue Kit (Qiagen) and was used for all subsequent analyses.

**Table 1:** Details of which molecular analyses were conducted with *A. hymenopus* collected from each study site

Site	Microbiome	*Bd* infection	Population genetics
Khubnam			●
Vemvane			●
Nampolice	●	●	●
Tugela	●	●	●
Tugela 2	●	●	●
Tukelahed	●	●	●
Bilanjil	●	●	●
Ribbon Falls	●	●	●

### *Bd* infection

We constructed serial dilutions of DNA and conducted quantitative PCRs on a Qiagen Rotor Gene according to Boyle et al. [25]. We quantified *Bd* infection intensity in triplicate using a 1:10 dilution of DNA with *Bd* standards ranging from 0.025 to 250 zoospores/µl. We calculated infection prevalence and intensity for each site.

### 16S amplicon sequencing of bacterial communities

We sequenced the microbial communities present on tadpole mouthparts using a modified version of the protocol in Kozich et al. and Bates et al. [26, 27]. Transient bacteria most likely rinsed off during the ethanol preservation and so microbial analyses reflect the resident microbiome in the mouthparts of tadpoles. Briefly, we amplified a V4 region of the 16S rRNA gene in triplicate for 139 samples, as well as two negative controls (one per PCR plate). Triplicates were pooled and normalized using a titration run on an Illumina MiSeq using v2 nano chemistry, followed by a full run using 250 bp paired-end v2 chemistry. We also sequenced a mock community containing 20 bacteria isolated from amphibian skin (genera *Acinetobacter, Citrobacter, Enterobacter*, *Flavobacterium, Plantibacter, Pseudomonas* and *Serratia*).

#### Analyses

We performed sequence processing in DADA2 v1.4.0 (Calahan et al., 2016) using the default pipeline (see Supplementary Information). Modal contig length was 253 bp once paired-end reads were merged. We filtered 145 sequence variants (SVs) with length >260 bp (0.65% of total sequences), and excluded 190 bimeras from the 22,010 input sequences. We assigned taxonomy using the SILVA v123 training set [28, 29]. We removed 41 contaminating SVs found in the negatives, including several variants of halophilic bacteria, such as *Halomonas* and *Pseudoalteromonas* that are likely PCR mastermix contaminants. DADA2 identified 20 unique sequence variants in the sequenced mock community sample comprising 20 bacterial isolates. The final SV table, taxonomy table, and sample metadata were exported to *phyloseq* [30] in R v3.3.3 [31].

Following Longo and Zamudio [32], we excluded SVs that contained fewer than 100 reads across the entire dataset (*n* = 19,395), yielding 2384 SVs. Results using all SVs are similar and not shown here. After filtering, mean library size across individuals was 20,160 reads (range 13,156–83,063). We rarefied all libraries to 13,156 reads per sample for alpha and beta diversity analyses. We calculated alpha diversity metrics (Shannon index) using the ‘estimate_richness’ command in *phyloseq*. We visualised differences in Bray-Curtis distance among samples using non-metric multi-dimensional scaling (NMDS) ordination of bacterial communities using the R package *vegan* [33], setting *k* to 3 to yield a stress value of <0.17. We also used the *vegan* function ‘vegdist’ to calculate Bray-Curtis distance between bacterial communities to compare to genetic distances among samples, and the ‘adonis’ function to compare bacterial community structure among populations using permutational MANOVA. We used the R package *gplots* [34] to visualize the number of shared SVs among populations with a Venn diagram. To simplify the Venn diagram, we collapsed the two ‘Tugela’ sampling points into one group.

### Population genetics

#### Microsatellite marker development and genotyping

We sequenced DNA from a single *A. hymenopus* individual using Illumina 250 bp paired-end v2 chemistry. We characterised eleven novel tri- and tetra-nucleotide microsatellite markers according to Griffiths et al. [35] (Supplementary Information, Table S1). We conducted PCRs in five multiplex reactions with the Type-it® Microsatellite Kit (Qiagen) with the following conditions: 95 °C for 5 min; 30–35 × 95 °C for 30 s, 60–66 °C for 90 s, 72 °C for 30 s; 60 °C for 30 min (Table S1). We sized PCR amplicons using the 3730 DNA Analyzer and the GeneScan™ LIZ1200 size standard (Thermo-Fisher Scientific), scored alleles in Genemapper v.3.7 (Thermo-Fisher Scientific), and binned alleles in MsatAllele v1.02 [36] in R v3.3.3 [31].

#### Analyses

Details of quality control and summary statistics carried out can be found in  Supplementary Information. To assess levels of population differentiation, we calculated global *F*_ST_ and pairwise *F*_ST_ values between sites with corrections for null alleles using the ENA method of Chapuis and Estoup [37] with 1000 bootstrap replicates, and pairwise *D* [38] in Genodive v2.0b23. [39] We used ADZE v1.0 [40] to calculate site-level rarefied allelic richness (maximum *g* = 20) to study the effects of intraspecific genetic diversity at the population level on microbiome variation. We excluded Khubnam due to the low sample size (*n* = 9) compared with other sites (*n* = 20–28) to ensure sufficient power to detect differences among sites was retained. We created a pairwise matrix of Bruvo genetic distance [41] between all individuals in GenoDive. We also calculated individual-level heterozygosity (proportion of heterozygous loci in all loci successfully genotyped) using Genhet [42] in R to study its effect on associated microbial communities, as individual heterozygosity can be correlated with fitness.

We used Bayesian clustering program STRUCTURE v2.3.4 [43, 44] to assess the number of population clusters (*K*) and assign individuals to them based on likelihoods. We inferred the most probable *K* using the Evanno method [45, 46], and used CLUMPP v1.1.2 [47] and Distruct v1.1 [48] to process the output.

We conducted principal coordinates analysis (PCoA) in GenAlEx v6.502 [49, 50] using pairwise *D* values and the standardised covariance method. We ran an analysis of molecular variance (AMOVA) in Genodive using the Infinite Allele Model. We tested for the presence of isolation by distance among all locations and within population clusters. We conducted a Mantel test in *ade4* in R [51] in R with 999 permutations to test associations between matrices of pairwise linearised *F*_ST_ values (*F*_ST_/1–*F*_ST_) and the logarithm of geographic distances. We used GENECLASS2 v2.0 [52] to identify putative first-generation migrants among sites [53, 54].

We used INEST v2.1 [55] to identify the presence of two genetic signatures of bottleneck events; heterozygosity excesses in respect to allelic richness [56] and deficiency in M-Ratio values (mean ratio of the number of alleles to the range in allele size; [57]).

### Predictors of microbial community structure

#### Individual level data

To examine the factors affecting Shannon diversity, we fitted a Gaussian Mixed effects model in the R package *lme4* [58] with Shannon index as the response, Gosner stage and heterozygosity (specified as proportion of heterozygous loci, [59]) as predictors, and population ID as a random intercept (*n* = 126 data points for which there were both microbial (Shannon) and host genetic (proportion of heterozygous loci) data). Gosner stage and proportion of heterozygous loci were *z*-transformed prior to model fitting. The most complex model contained a proportion of heterozygous loci × Gosner stage interaction. We assessed significance of terms using likelihood ratio tests between models estimated using maximum likelihood. To quantify differences in mean richness among populations, we extracted the posterior modes of the population random effects from a Bayesian version of the best-supported model, specified in the R package *MCMCglmm* [60].

To examine the factors predicting microbial community structure (beta diversity), we calculated the within-population ‘divergence’ metric of beta diversity using the *microbiome* package (Lahti et al. 2017) to be used as a response in a Gaussian mixed effects model. The community structure model contained Gosner stage, proportion of heterozygous loci and their interaction as fixed effects, and population ID as a random effect. We calculated the *r*^2^ values of minimal models using the [61] method for mixed effects models, as implemented in the R package *MuMIn* [62]. We used a partial Mantel test to examine the correlation between genetic distances among *A. hymenopus* individuals (calculated from microsatellite data) and Bray-Curtis dissimilarities among their microbial communities (calculated from 16S amplicon data), while controlling for the effect of geographic distance. We used the ‘mantel.partial’ function in the *vegan* package, specifying the Pearson correlation statistic.

#### Population level data

We fitted population-level regressions (*n* = 6 sampling sites) to test the influence of allelic richness and *Bd* infection prevalence on alpha diversity. We did not fit both *Bd* prevalence and allelic richness in the same model to maximize residual degrees of freedom in the model. Significance of terms was assessed by likelihood ratio test.

## Results

### *Bd* infection

The average *Bd* infection intensity across all individuals was 0.19 (±0.02) zoospores, with an average prevalence of 10.91 (±3.92)% across populations. There was relatively low infection prevalence and intensity at all sites, with highest infection rates at Tugela 2 and a complete absence of infection at Ribbon Falls (Table 2).

**Table 2:** Summary results of qPCR analyses of *Batrachochytrium dendrobatidis* infection of *Amietia hymenopus*

Site	Number of individuals sampled	Number of infected individuals	Infection prevalence (%)	Average infection intensity (zoospore equivalents)	Range (zoospore equivalents)
Nampolice	21	1	4.76	0.07	0.00–1.50
Tugela	24	2	8.33	0.02	0.00–0.50
Tugela 2	21	5	23.81	0.89	0.00–15.73
Tukelahed	20	1	5.00	0.01	0.00–0.01
Bilanjil	23	4	17.39	0.22	0.00–4.53
Ribbon Falls	25	0	0.00	0.00	0.00–0.00

### Microbiome structure

**Fig. 2:**
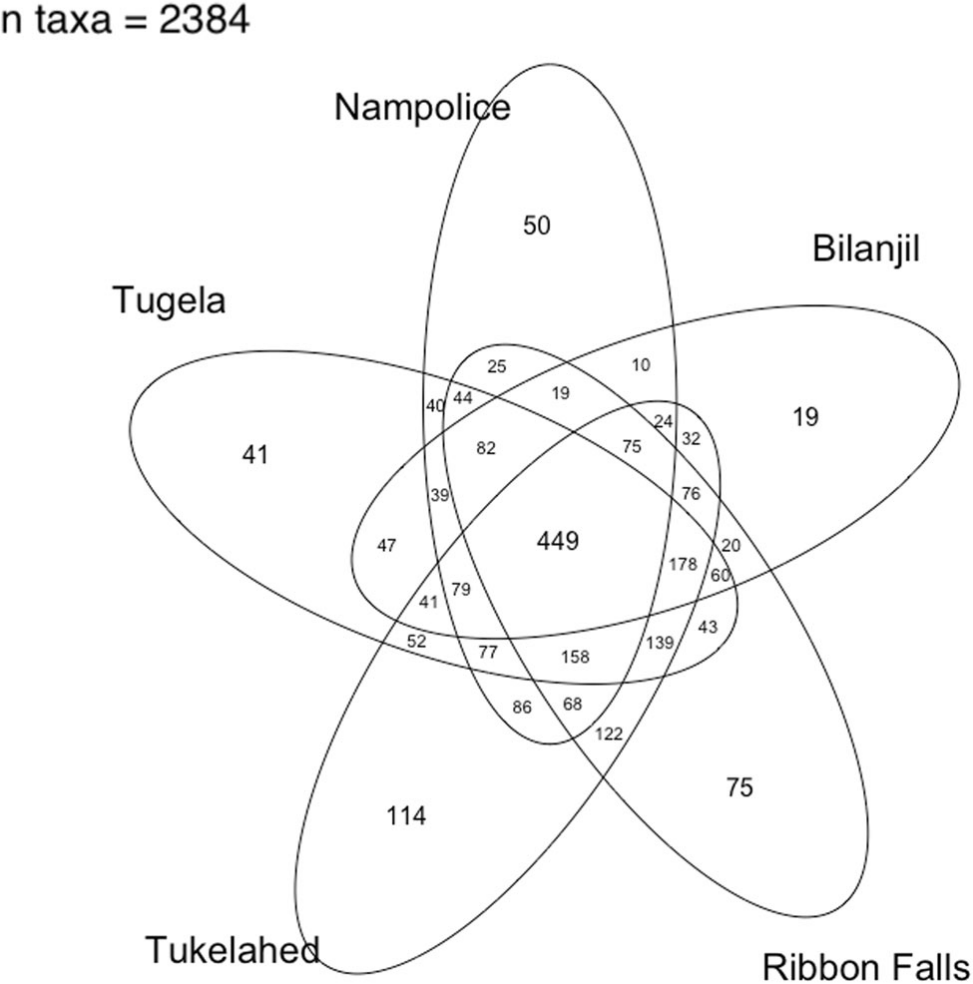
Venn diagram illustrating unique and shared sequence variants (SVs) by sampling location. Here the two Tugela sampling locations have been collapsed into one group for ease of presentation

Of the 2384 SVs in the filtered dataset, 449 SVs (18.83%) were common to all sampling sites (Fig. 2). Tukelahed had the highest number of unique SVs (114), followed by Ribbon Falls (75) and Nampolice (50). Bacterial communities were dominated at the family level by Cytophagaceae, Comamonadaceae, Saprospiraceae, Flavobacteraceae and Chitinophageaceae.

**Fig. 3:**
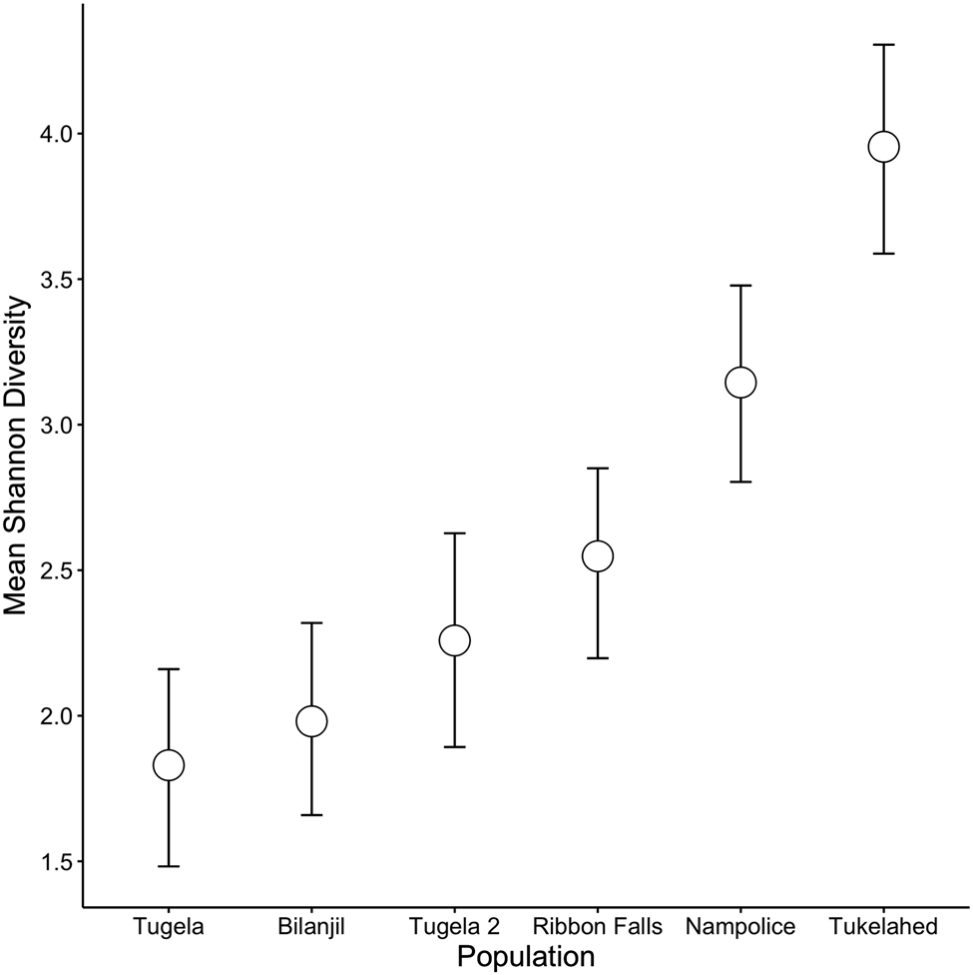
Mean Shannon diversity by site for each of the six sampling locations. Points are posterior mean estimates of a Bayesian model of the effect of site identity on Shannon diversity. Bars are 95% credible intervals

**Fig. 4:**
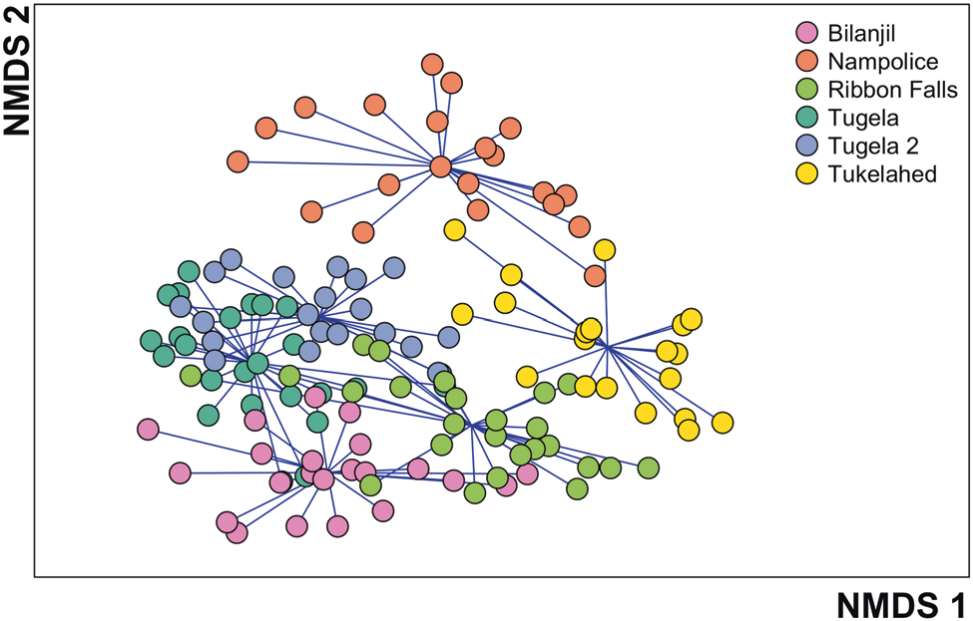
Non-metric multi-dimensional scaling (NMDS) ordination of bacterial communities on tadpole mouthparts. Colours indicate different sampling sites. There was significant differentiation among sampling sites in bacterial community structure

We observed significant differences in Shannon diversity index among sampling sites (Fig. 3). Tukelahed had the highest alpha diversity, whilst the values for the remaining five sampling locations were significantly lower (95% credible intervals of difference all >0; Fig. 3). There was no significant difference in the alpha diversity of the two Tugela sampling locations (95% credible interval of difference −0.92 to 0.05). NMDS ordination revealed distinct separation of bacterial community centroids corresponding to sampling site (Fig. 4). PERMANOVA of community distances supported this pattern, with significant separation among sampling sites (*F*_5,127_ = 18.64, *r*^2^ = 42.32%; *p* < 0.001).

Shannon diversity of tadpole mouthparts was not affected by either Gosner stage of tadpoles (*χ*^2^ = 2.61, *p* = 0.1), proportion of heterozygous loci (*χ*^2^ = 2.19, *p* = 0.14), or their interaction (*χ*^2^ = 0.19, *p* = 0.67).

**Fig. 5:**
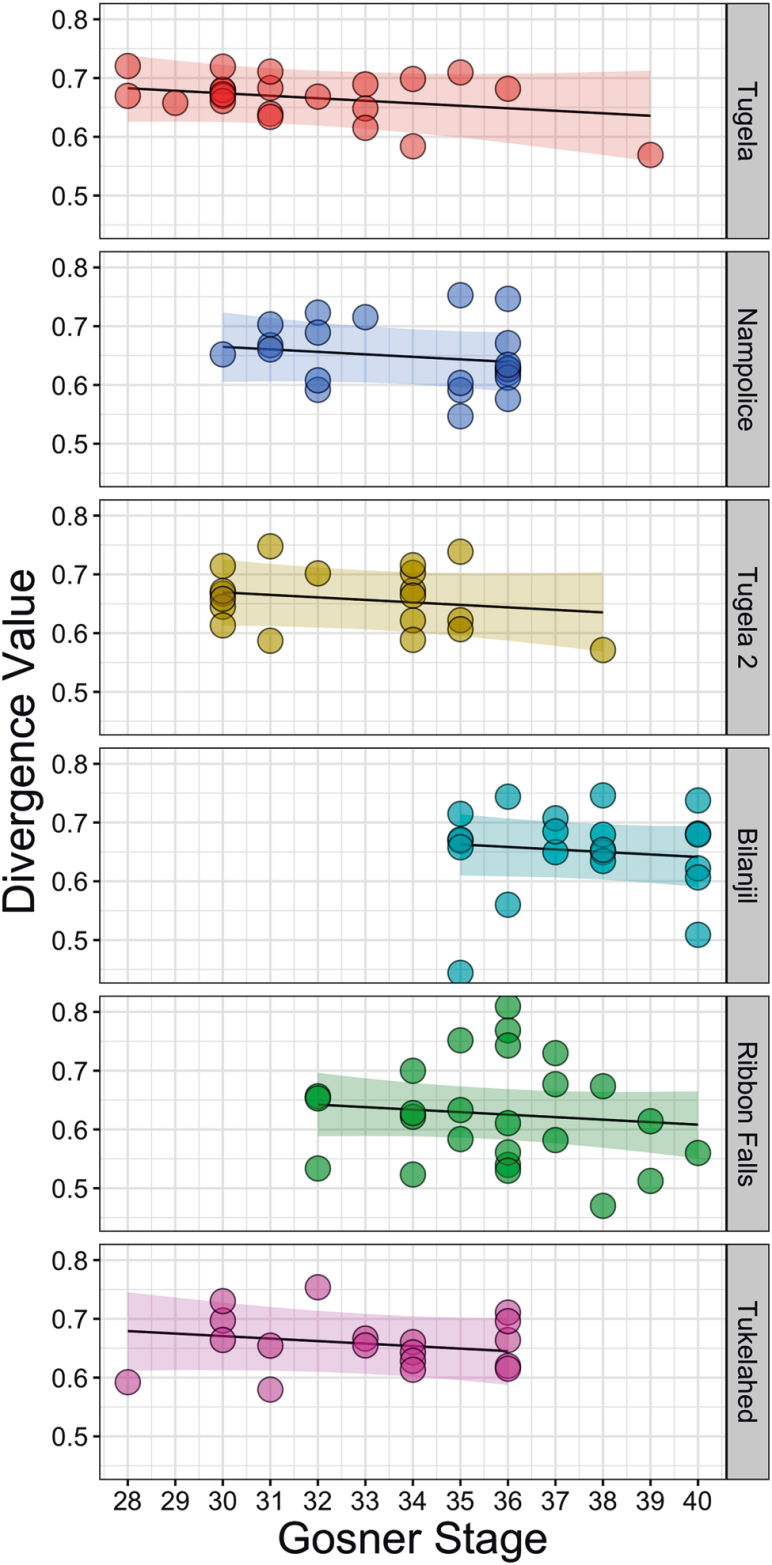
Divergence values of beta diversity decreased significantly with Gosner stage of tadpoles across sampling sites. Lines are the posterior modes of the relationship between age and divergence, conditioned on sampling site ID. Shaded areas around lines represent 95% credible intervals. Points are raw data, coloured by sampling site

Gosner stage significantly influenced within-population divergence in beta diversity (*χ*^2^ = 5.01, *p* = 0.025; Fig. 5). There was no evidence of an effect of an interaction between Gosner stage and proportion of heterozygous loci on beta diversity, or proportion of heterozygous loci as a main effect (all *p* > 0.47). The best model explaining differences in beta diversity contained Gosner stage as the only fixed effect and had a marginal *r*^2^ of 4%.

There was a positive correlation between host genetic distance calculated from microsatellites and microbial community distance when controlling for geographic distance (Partial Mantel Test *r* = 0.145, *p* < 0.001). There was no evidence that the Shannon diversity at a sampling site was affected by site-level allelic richness (*F*_1,4_ = 0.95, *p* = 0.38) or *Bd* infection prevalence (*F*_1,4_ = 1.17, *p* = 0.34).

### Population genetics

**Fig. 6:**
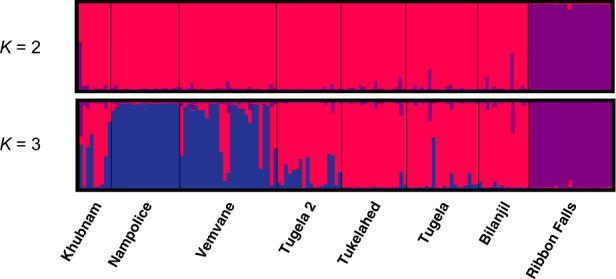
Bayesian population assignment analysis STRUCTURE plots showing membership coefficients (*Q*) for *K* = 2 and *K* = 3 genetic clusters in *Amietia hymenopus*. Vertical bars represent individuals, and colours represent a genetic cluster; the height of the bar represents the proportion of the genotype assigned to that cluster

**Fig. 7:**
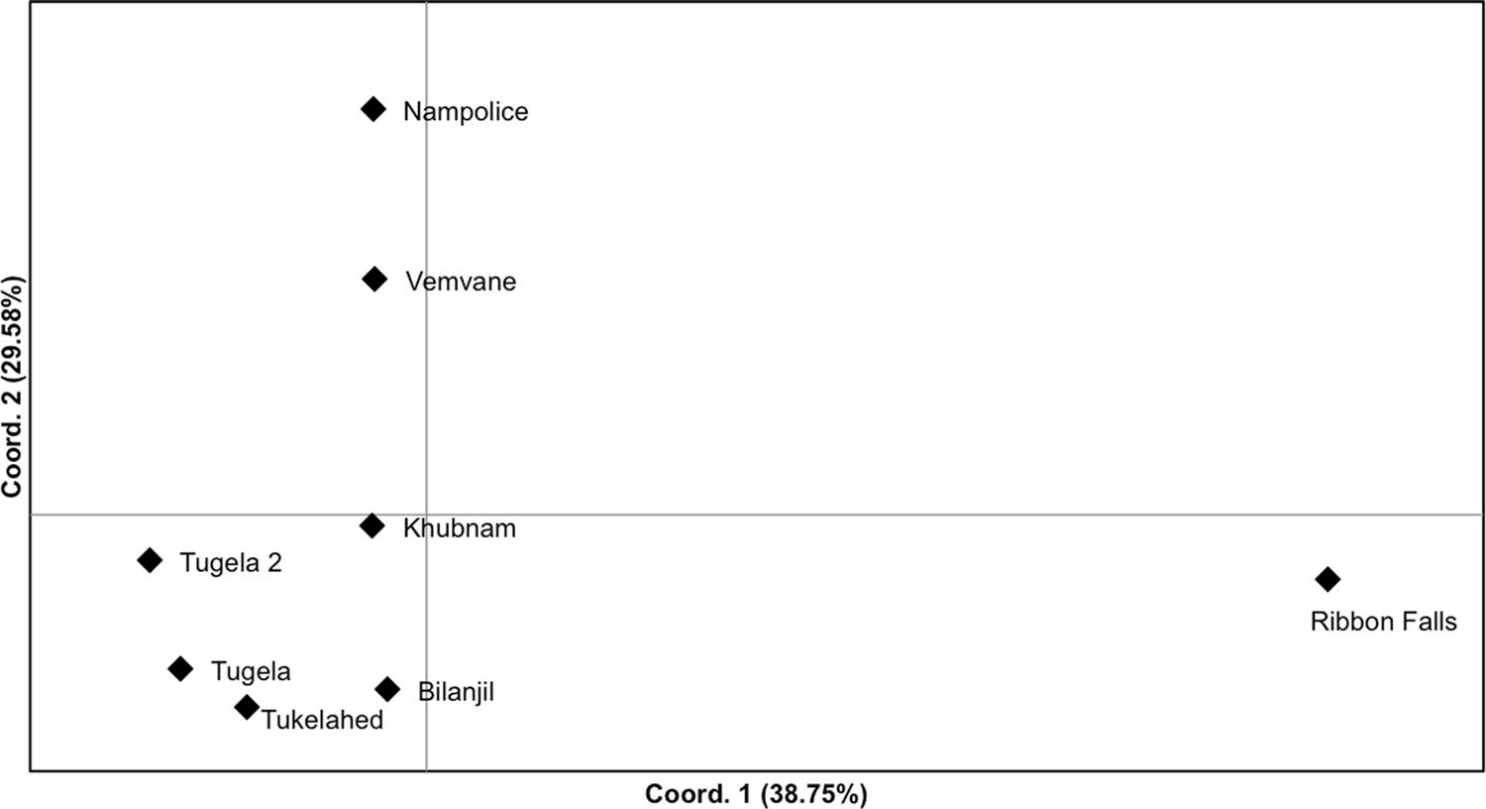
Principal coordinates analysis (PCoA) of pairwise *D* [38] estimates among *Amietia hymenopus* sites. Percentages show the amount of variance explained by each coordinate

Strong genetic structure was found in *A. hymenopus*, with *F*_ST_ levels reaching 0.292 between Ribbon Falls and Nampolice (Table S2 and Supplementary Information). Ribbon Falls was highly differentiated from all sites, and in general sites from different rivers showed moderate levels of genetic differentiation, while sites within the same river showed low differentiation. STRUCTURE showed the optimum *K* to be two (Fig. 6), with Ribbon Falls forming a distinct population cluster and the remaining sites another. At *K* = 3, Vemvane and Nampolice also formed a separate cluster (Fig. 6). Hierarchical clustering analysis conducted on the population cluster of the *K* = 2 analysis (i.e., repeating the STRUCTURE run without Ribbon Falls) supported the presence of a Nampolice and Vemvane cluster. The PCoA and AMOVAs showed results consistent with the STRUCTURE analysis (Fig. 7, Table S4), and together indicate that Ribbon Falls is a distinct population cluster, and Vemvane and Nampolice form a subpopulation cluster within the cluster of remaining sites.

Isolation by distance was strong and significant when tested over all locations (*p* = 0.002, *r* = 0.675), and within the population cluster identified in STRUCTURE *K* = 2 (i.e. Khubnam, Nampolice, Vemvane, Tugela, Tugela 2, Tukelahed and Bilanjil) (*r* = 0.598, *p* = 0.024). However, when tested within the Khubnam, Tugela, Tugela 2, Tukelahed and Bilanjil group identified in the PCoA and STRUCTRE *K* = 3, isolation by distance was not significant (*r* = 0.694, *p* = 0.054). Four putative first-generation migrants were found among the site: One in Nampolice originating from Vemvane (*p* = 0.009), one migrant each were exchanged between Tugela 2 and Bilanjil (Bilanjil to Tugela 2: *p* < 0.001; Tugela 2 to Bilanjil: *p* = 0.006), and one in Tukelahed originating from Tugela 2 (*p* = 0.003).

Bottlenecks were detected at three sites: Heterozygosity excess in respect to allelic richness was found at Nampolice (*p* = 0.003), and deficiencies in M-ratios were found at Nampolice (*p* = 0.003), Vemvane (*p* < 0.001) and Ribbon Falls (*p* = 0.042).

## Discussion

Population structure in *A. hymenopus* was strongly influenced by river network and geographic distance. However, at the landscape level, the microbiome of *A. hymenopus* is not structured according to host population genetic structure or river system. For example, Tukelahed did not have a similar microbial community structure to downstream sites Tugela and Tugela 2 (Fig. 4). Despite a lack of similarity between host population genetic structure and site-level microbiome structure, genetic distance among hosts is still significantly correlated with associated microbial community dissimilarity when controlling for geographic distance. This has not been previously shown in amphibians, but host genotype influences the gut microbiota of chickens [16], mice [63] and fish [7], and inbreeding and relatedness (although not population genetic structure) influence gut microbiomes of gopher tortoises (*Gopherus polyphemus*; [8]). Although not tested here, these patterns may be due to genetic variation in microsatellites reflecting variation in phenotypic traits that affect microbiome composition. Microsatellites can be linked to genes coding for functional traits [64–66], including immune response genes in a number of species [67–69]. Variation in these genes could influence the composition of microbiomes by controlling the horizontal transmission of microbes from the environment. In *A. hymenopus*, the microbiome of the tadpole mouthparts is most likely transmitted from the environment as they are feeding or behaviourally selecting areas in the river. Behaviours can also influence symbiont transmission [70], and therefore there may be a link between genetic variation, behavioural variation, and microbe acquisition. That the influence of host genotype does not extend to landscape level microbiome structure may indicate that site-level environmental differences have more influence in structuring tadpole microbiomes, and host genotype simply exerts some selection pressure or behavioural influence on horizontal transmission.

We detected a link between Gosner stage and mouthpart microbiome composition. Studies to date have predominantly used skin swabs to determine host microbiome of amphibians, as this is the region that becomes infected by *Bd* on post-metamorphic individuals. However, *Bd* primarily infects the mouthparts of larvae as keratin is localised to this area until the onset of metamorphosis when skin is remodelled to contain keratin. Although *Bd* infection intensities tend to increase as larvae develop due to increasing size [71], we show that alpha diversity (i.e., number of SVs) of the larval mouthpart microbiome does not increase as Gosner stage increases. This indicates that ontogenetic variation in microbiome is not simply an artefact of increasing mouthpart size, but rather it is the specific composition of the community that is changing as tadpoles develop. These microbiome changes may be related to phenotypic changes in mouthparts over development [24, 72], which occur in other montane river species in the region during development as an adaptation to fast-flowing water [73]. Ontogenetic variation may also arise from changes in behaviour throughout larval development that means individuals are exposed to different environmental conditions and thus, microbial influences; however, this has not been studied in this species.

Despite these genotypic and ontogenetic influences on microbiome composition, populations shared a considerable core microbiome (Fig. 2), which has also been shown for other amphibian species [6, 74], including among genetically isolated populations [75]. Our population genetics analyses detected low numbers of first-generation migrants between populations, with high-genetic differentiation given the relatively small geographic area studied. Together, these results indicate very low levels of dispersal among rivers, and in some instances, low dispersal among tributaries in the same river. This implies that core microbiome observed across the metapopulation of *A. hymenopus* has not arisen from contact between populations. A number of studies have now indicated that amphibians are able to select their microbiome based on functional traits [76, 77]. This may be related to conserved host traits, such as anti-microbial peptide composition, or adaptive genetic loci. In addition, the river system and associated vegetation is remarkably homogenous on the uninhabited and unspoiled ecosystem of the Mont-aux-Sources plateau, with very low diversity of plants [78]. This may also in part explain the shared microbiome exhibited across populations. That said, there is likely to be fine-scale variation between environmental conditions in the rivers, which may be driving some variation in microbiome composition [6, 79, 80]. Microbial communities have been shown to vary between rivers even when colonisation substrate is controlled for [81]. Individuals from Nampolice and Tukelahed had the most distinct microbiome (Fig. 4), and the highest alpha diversity (Fig. 3), and this may be related to proximity from the source of the river. For example, Górniak et al [82] found bacterial diversity generally decreased as they sampled downstream from the source (glacial lake), and linked this to changes in environmental conditions and nutrient availability.

Historically there is wide annual and geographical variation in *Bd* infection prevalence in the Mont-aux-Sources region, ranging from ~0 to 100% [23]. Although, we found uncharacteristically low infection loads in this study, our data correspond to historical patterns for the three sites that have undergone long-term monitoring. Ribbon Falls has historically low infection prevalence, followed by Bilanjil, and then Tugela, which shows the highest infection prevalence [23]. This may be driven by a range of environmental factors similar to those that drive microbiome diversity, including water flow, temperature, pH and depth [71]. Particularly low infection intensities such as those found here are, however, difficult to assess accurately using quantitative PCR. Given these low infection intensities, it is not possible to identify meaningful relationships between *Bd* infection and microbiome diversity, although this has been demonstrated in other studies ([2, 12, 27, 76, 83, 84]).

Ribbon Falls, Nampolice and Vemvane showed evidence of recent population bottlenecks, and these sites also have some of the lowest allelic richness. Considerable mortalities of *A. hymenopus* were seen at Ribbon Falls and Vemvane in September 2006 during routine monitoring (every 2–4 months) of Vemvane, Tugela, Bilanjil and Ribbon Falls ([23]; C. Weldon, pers. obvs.). Diagnostic analyses identified *Bd* infections in 100% of the dead metamorphic *A. hymenopus* (*n* = 10) collected at each site. Further dead post-metamorphic individuals (*n* = 1–12) were encountered on most of the subsequent surveys, all infected with *Bd* [23]. Low genetic diversity in mortality-affected sites is unsurprising given the low level of migration from other sites. This presents a potential danger in bottlenecked locations struggling to recover evolutionary potential, impacting their ability to respond to stressors such as climate change, disease or local climatic events. Local chytrid-induced mass mortality events may eliminate genetic diversity from the species as a whole due to the distinct population genetic structure and low migration. Interestingly, *Bd* infection prevalence at Ribbon Falls is generally low compared to other rivers in the region (this study and [23]), and Ribbon Falls has the lowest genetic diversity, which may be because of selection of *Bd*-resistant individuals. Addis et al. [85] showed montane boreal toads (*Bufo boreas*) with higher heterozygosity had higher *Bd* infection intensity, which they attribute to increased dispersal of heterozygous individuals leading to increased spread of *Bd*. Therefore, it is possible that the lack of immigrants to Ribbon Falls is also limiting the spread and maintenance of *Bd* at this site.

## Conclusion

Here we have shown that the microbiome composition of amphibians is determined by genetic and ontogenetic variation, as well as geographic site, indicating within and among population predictors of microbiome diversity. *Bd* prevalence was low in the year we sampled, but we found lower genetic diversity and signals of genetic bottlenecks in sites where mass mortalities have been recorded in previous years, demonstrating the vulnerability to and persisting effects of disease in amphibian populations. Together these results demonstrate the key role of host genotype on microbial colonisation, which may be important in pathogen susceptibility in the amphibian-chytridiomycosis system. Identifying individuals based on this characteristic may aid amphibian conservation efforts against this lethal pathogen.

## Electronic supplementary material

Supplementary material

## References

[1] Cariveau DP, Powell JE, Koch H, Winfree R, Moran NA (2014). Variation in gut microbial communities and its association with pathogen infection in wild bumble bees (*Bombus*). ISME J.

[2] Jani AJ, Briggs CJ (2014). The pathogen batrachochytrium dendrobatidis disturbs the frog skin microbiome during a natural epidemic and experimental infection. Proc Natl Acad Sci USA.

[3] Theriot CM, Koenigsknecht MJ, Carlson PE, Hatton GE, Nelson AM, Li B (2014). Antibiotic-induced shifts in the mouse gut microbiome and metabolome increase susceptibility to *Clostridium difficile* infection. Nat Commun.

[4] Jani AJ, Knapp RA, Briggs CJ (2017). Epidemic and endemic pathogen dynamics correspond to distinct host population microbiomes at a landscape scale. Proc R Soc.

[5] Krynak KL, Burke DJ, Benard MF (2016). Landscape and water characteristics correlate with immune defense traits across Blanchard’s cricket frog (*Acris blanchardi*) populations. Biol Conserv.

[6] Kueneman JG, Parfrey LW, Woodhams DC, Archer HM, Knight R, McKenzie VJ (2013). The amphibian skin-associated microbiome across species, space and life history stages. Mol Ecol.

[7] Bolnick DI, Snowberg LK, Caporaso JG, Lauber C, Knight R, Stutz WE (2014). Major histocompatibility complex class iib polymorphism influences gut microbiota composition and diversity. Mol Ecol.

[8] Yuan ML, Dean SH, Longo AV, Rothermel BB, Tuberville TD, Zamudio KR (2015). Kinship, inbreeding and fine-scale spatial structure influence gut microbiota in a hindgut-fermenting tortoise. Mol Ecol.

[9] Garner TWJ, Schmidt BR, Martel A, Pasmans F, Muths E, Cunningham AA (2016). Mitigating amphibian chytridiomycoses in nature. Philos Trans R Soc Lond B Biol Sci.

[10] Kueneman JG, Woodhams DC, Harris R, Archer HM, Knight R, McKenzie VJ (2016). Probiotic treatment restores protection against lethal fungal infection lost during amphibian captivity. Proc Biol Sci.

[11] Rebollar EA, Hughey MC, Medina D, Harris RN, Ibáñez R, Belden LK (2016). Skin bacterial diversity of panamanian frogs is associated with host susceptibility and presence of batrachochytrium dendrobatidis. ISME J.

[12] Becker MH, Walke JB, Murrill L, Woodhams DC, Reinert LK, Rollins-Smith LA (2015). Phylogenetic distribution of symbiotic bacteria from panamanian amphibians that inhibit growth of the lethal fungal pathogen batrachochytrium dendrobatidis. Mol Ecol.

[13] Blekhman R, Goodrich JK, Huang K, Sun Q, Bukowski R, Bell JT (2015). Host genetic variation impacts microbiome composition across human body sites. Genome Biol.

[14] Lundberg DS, Lebeis SL, Paredes SH, Yourstone S, Gehring J, Malfatti S (2012). Defining the core arabidopsis thaliana root microbiome. Nature.

[15] Wagner MR, Lundberg DS, Del Rio TG, Tringe SG, Dangl JL, Mitchell-Olds T (2016). Host genotype and age shape the leaf and root microbiomes of a wild perennial plant. Nat Commun.

[16] Zhao L, Wang G, Siegel P, He C, Wang H, Zhao W (2013). Quantitative genetic background of the host influences gut microbiomes in chickens. Sci Rep.

[17] Woodhams DC, Rollins-Smith LA, Carey C, Reinert L, Tyler MJ, Alford RA (2006). Population trends associated with skin peptide defenses against chytridiomycosis in Australian frogs. Oecologia.

[18] Bresciano JC, Salvador CA, Paz-y-Mino C, Parody-Merino AM, Bosch J, Woodhams DC (2015). Variation in the presence of anti- *Batrachochytrium dendrobatidis* bacteria of amphibians across life stages and elevations in Ecuador. Ecohealth.

[19] Sanchez E, Bletz MC, Duntsch L, Bhuju S, Geffers R, Jarek M (2017). Cutaneous bacterial communities of a poisonous salamander: a perspective from life stages, body parts and environmental conditions. Microb Ecol.

[20] Briggs CJ, Knapp RA, Vredenburg VT (2010). Enzootic and epizootic dynamics of the chytrid fungal pathogen of amphibians. Proc Natl Acad Sci USA.

[21] Smith KG, Weldon C, Conradie W, Du Preez LH (2007). Relationships among size, develoment, and Batrachochytrium dendrobatidis infection in African tadpoles. Dis Aquat Org.

[22] Channing A (2015). The maluti mystery revisited: taxonomy of African river frogs (Pyxicephalidae, Amietia) on the Drakensberg Mountains in southern Africa. Zootaxa.

[23] Pretorius A. Disease dynamics in a metapopulation of *Amietia hymenopus*. Masters Thesis submitted to North West University, Potchefstroom, South Africa, 2016.

[24] Gosner KL (1960). A simplified table for staging anuran embryos and larvae with notes on identification. Herpetologica.

[25] Boyle DG, Boyle DB, Olsen V, Morgan JA, Hyatt AD (2004). Rapid quantitative detection of chytridiomycosis (Batrachochytrium dendrobatidis) in amphibian samples using real-time taqman PCR assay. Dis Aquat Organ.

[26] Kozich JJ, Westcott SL, Baxter NT, Highlander SK, Schloss PD (2013). Development of a dual-index sequencing strategy and curation pipeline for analyzing amplicon sequence data on the MiSeq Illumina sequencing platform. Appl Environ Microbiol.

[27] Bates KA, Clare FC, O’Hanlon S, Bosch J, Brookes L, Hopkins K, et al. Amphibian chytridiomycosis outbreak dynamics are linked with host skin bacterial community structure. Nat Commun. 2018;9:693. 10.1038/s41467-018-02967-w PMC5814395 29449565

[28] Pruesse E, Quast C, Knittel K, Fuchs BM, Ludwig WG, Peplies J (2007). SILVA: a comprehensive online resource for quality checked and aligned ribosomal RNA sequence data compatible with ARB. Nucleic Acids Res.

[29] Quast C, Pruesse E, Yilmaz P, Gerken J, Schweer T, Yarza P (2013). The SILVA ribosomal RNA gene database project: Improved data processing and web-based tools. Nucleic Acids Res.

[30] McMurdie P, Holmes S (2013). phyloseq: an R package for reproducible interactive analysis and graphics of microbiome census data. PLoS ONE.

[31] R Core Team. R: a language and environment for statistical computing. R Foundation for Statistical Computing, Vienna, Austria. 2017. URL https://www.R-project.org/.

[32] Longo AV, Zamudio KR (2017). Temperature variation, bacterial diversity and fungal infection dynamics in the amphibian skin. Mol Ecol.

[33] Oksanen J, Blanchet FG, Friendly M, Kindt R, Legendre P, McGlinn D et al. vegan: Community Ecology Package. R package version 2.4-3. 2017 https://CRAN.R-project.org/package=vega

[34] Warnes GR, Bolker B, Bonebakker L, Gentleman R, Huber W, Liaw A et al. gplots: various R programming tools for plotting data. R package version, 2. 2009.

[35] Griffiths SM, Fox G, Briggs PJ, Donaldson IJ, Hood S, Richardson P (2016). A galaxy-based bioinformatics pipeline for optimised, streamlined microsatellite development from Illumina next-generation sequencing data. Conserv Genet Resour.

[36] Alberto F (2009). MsatAllele_1.0: an R package to visualize the binning of microsatellite alleles. J Hered.

[37] Chapuis MP, Estoup A (2007). Microsatellite null alleles and estimation of population differentiation. Mol Biol Evol.

[38] Jost L (2008). GST and its relatives do not measure differentiation. Mol Ecol.

[39] Meirmans PG, Van Tiendener PH (2004). Genotype and Genodive: two programs for the analysis of genetic diversity of asexual organisms. Mol Ecol Notes.

[40] Szpiech ZA, Jakobsson M, Rosenberg NA (2008). ADZE: a rarefaction approach for counting alleles private to combinations of populations. Bioinformatics.

[41] Bruvo R, Michiels NK, D’Souza TG, Schulenburg H (2004). A simple method for the calculation of microsatellite genotype distances irrespective of ploidy level. Mol Ecol.

[42] Coulon A (2010). genhet: an easy-to-use R function to estimate individual heterozygosity. Mol Ecol Res.

[43] Falush D, Stephens M, Pritchard JK (2007). Inference of population structure using multilocus genotype data: dominant markers and null alleles. Mol Ecol Res.

[44] Pritchard JK, Stephens M, Donnelly P (2000). Inference of population structure using multilocus genotype data: dominant markers and null alleles. Genetics.

[45] Earl DA, vonHoldt BM (2011). STRUCTURE HARVESTER: a website and program for visualizing STRUCTURE output and implementing the Evanno method. Conserv Genet Resour.

[46] Evanno G, Regnaut S, Goudet J (2005). Detecting the number of clusters of individuals using the software STRUCTURE: a simulation study. Mol Ecol.

[47] Jakobsson M, Rosenberg NA (2007). CLUMPP: a cluster matching and permutation program for dealing with label switching and multimodality in analysis of population structure. Bioinformatics.

[48] Rosenberg NA (2004). distruct: a program for the graphical display of population structure. Mol Ecol Res.

[49] Peakall R, Smouse PE (2006). genalex 6: genetic analysis in Excel. Population genetic software for teaching and research. Mol Ecol Notes.

[50] Peakall R, Smouse PE (2012). GenAlEx 6.5: genetic analysis in Excel. Population genetic software for teaching and research--an update. Bioinformatics.

[51] Dray S, Dufour AB (2007). The ade4 package: implementing the duality diagram for ecologists. J Stat Softw.

[52] Piry S, Alapetite A, Cornuet JM, Paetkau D, Baudouin L, Estoup A (2004). GENECLASS2: a software for genetic assignment and first-generation migrant detection. J Hered.

[53] Paetkau D, Slade R, Burden M, Estoup A (2004). Genetic assignment methods for the direct, real-time estimation of migration rate: a simulation-based exploration of accuracy and power. Mol Ecol.

[54] Rannala B, Mountain JL (1997). Detecting immigration by using multilocus genotypes. Proc Natl Acad Sci USA.

[55] Chybicki IJ, Burczyk J (2009). Simultaneous estimation of null alleles and inbreeding coefficients. J Hered.

[56] Cornuet JM, Luikart G (1996). Description and power analysis of two tests for detecting recent population bottlenecks from allele frequency data. Genetics.

[57] Garza JC, Williamson EG (2001). Detection of reduction in population size using data from microsatellite loci. Mol Ecol.

[58] Bates D, Maechler M, Bolker B (2015). Fitting linear mixed-effects models using lme4. J Stat Softw.

[59] Amos W, Worthington-Wilmer J, Fullard K, Burg TM, Croxall JP, Bloch D (2001). The influence of parental relatedness on reproductive success. Proc R Soc Lond B.

[60] Hadfield JD (2010). MCMC methods for multi-response generalized linear mixed models: the MCMCglmm R package. J Stat Softw.

[61] Nakagawa S, Schielzeth H (2013). A general and simple method for obtaining R2 from generalized linear mixed‐effects models. Methods Ecol Evol.

[62] Bartoń. MuMIn: multi-model inference. R package version 1.15.6. 2016. https://CRAN.R-project.org/package=MuMIn.

[63] Benson AK, Kelly SA, Legge R, Ma F, Low SJ, Kim J (2010). Individuality in gut microbiota composition is a complex polygenic trait shaped by multiple environmental and host genetic factors. Proc Natl Acad Sci USA.

[64] Gemayel R, Vinces MD, Legendre M, Verstrepen KJ (2010). Variable tandem repeats accelerate evolution of coding and regulatory sequences. Annu Rev Genet.

[65] Li YC, Korol AB, Fahima T, Nevo E (2004). Microsatellites within genes: structure, function, and evolution. Mol Biol Evol.

[66] Donaldson ZR, Kondrashov FA, Putnam A, Bai Y, Stoinski TL, Hammock EA (2008). Evolution of a behavior-linked microsatellite-containing element in the 5’ flanking region of the primate AVPR1A gene. BMC Evol Biol.

[67] Jensen LF, Hansen MM, Mensberg KLD, Loeschcke V (2008). Spatially and temporally fluctuating selection at non-MHC immune genes: evidence from TAP polymorphism in populations of brown trout (Salmo trutta, L.). J Hered.

[68] Santucci F, Ibrahim KM, Bruzzone A, Hewit GM (2007). Selection on MHC-linked microsatellite loci in sheep populations. J Hered.

[69] Tollenaere C, Ivanova S, Duplantier JM, Loiseau A, Rahalison L, Rahelinirina S (2012). Contrasted patterns of selection on MHC-linked microsatellites in natural populations of the malagasy plague reservoir. PLoS ONE.

[70] Ezenwa VO, Gerardo NM, Inouye DW, Medina M, Xavier JB (2012). Animal behavior and the microbiome. Science.

[71] Valencia-Aguilar A, Toledo LF, Vital MVC, Mott T (2016). Seasonality, environmental factors, and host behavior linked to disease risk in stream-dwelling tadpoles. Herpetologica.

[72] Altig R, McDiarmid, RW. Body Plan, development and morphology. In: McDiarmid, RW and Altig R editors. Tadpole: The biology of anuran larvae. The University of Chicago Press; 1999. p. 24–51.

[73] Conradie W, Conradie C (2015). Correlation between development and increase of number of labial tooth rows in Ghost Frog tadpoles (Anura: Heleophrynidae). Acta Herpetol.

[74] Loudon AH, Woodhams DC, Parfrey LW, Archer H, Knight R, McKenzie V (2012). Microbial community dynamics and effect of environmental microbial reservoirs on red-backed salamanders (plethodon cinereus). ISME J.

[75] Prado-Irwin Sofia R., Bird Alicia K., Zink Andrew G., Vredenburg Vance T. (2017). Intraspecific Variation in the Skin-Associated Microbiome of a Terrestrial Salamander. Microbial Ecology.

[76] Longo AV, Zamudio KR (2016). Environmental fluctuations and host skin bacteria shift survival advantage between frogs and their fungal pathogen. ISME J.

[77] Loudon AH, Venkataraman A, Van Treuren W, Woodhams DC, Parfrey LW, McKenzie VJ (2016). Vertebrate hosts as islands: dynamics of selection, immigration, loss, persistence, and potential function of bacteria on salamander skin. Front Microbiol.

[78] Killick DJB (1963). An account of the plant ecology of the Cathedral Peak area of the Natal Drakensberg. Botanical Survey Memoir.

[79] Fitzpatrick BM, Allison AL (2014). Similarity and differentiation between bacteria associated with skin of salamanders (plethodon jordani) and free-living assemblages. FEMS Microbiol Ecol.

[80] McKenzie VJ, Bowers RM, Fierer N, Knight R, Lauber CL (2011). Co-habiting amphibian species harbor unique skin bacterial communities in wild populations. ISME J.

[81] Marks JC, Haden GA, Harrop BL, Reese EG, Keams JL, Watwood ME (2009). Genetic and environmental controls of microbial communities on leaf litter in streams. Freshw Biol.

[82] Górniak D, Marszałek H, Jankowsk K, Dunalska J (2016). Bacterial community succession in an Arctic lake–stream system (Brattegg Valley, SW Spitsbergen). Boreal Environ Res.

[83] Walke Jenifer B., Becker Matthew H., Loftus Stephen C., House Leanna L., Teotonio Thais L., Minbiole Kevin P. C., Belden Lisa K. (2015). Community Structure and Function of Amphibian Skin Microbes: An Experiment with Bullfrogs Exposed to a Chytrid Fungus. PLOS ONE.

[84] Longo AV, Savage AE, Hewson I, Zamudio KR (2015). Seasonal and ontogenetic variation of skin microbial communities and relationships to natural disease dynamics in declining amphibians. R Soc Open Sci.

[85] Addis BR, Lowe WH, Hossack BR, Allendorf FW (2015). Population genetic structure and disease in montane boreal toads: more heterozygous individuals are more likely to be infected with amphibian chytrid. Conserv Genet.

