# Supplementary material for: Genetic variability and ontogeny predict microbiome structure in a disease-challenged montane amphibian
Source: ISME J. 2018 Jun 25;12(10):2506–17. doi: 10.1038/s41396-018-0167-0 (PMC6155040; doi:10.1038/s41396-018-0167-0)
Supplement: Supplementary file 1 — Supplementary material [file 41396_2018_167_MOESM1_ESM.pdf]

Supplementary information

GIS map construction

GPS locations of samples were recorded using a Garmin etrex 10 at an accuracy of ±3 m. Administrative boundary data for Lesotho and South Africa were obtained from the Global Administrative Database (GADM version 2.8; http://www.gadm.org/). Advanced Spaceborne Thermal Emission and Reflection Radiometer (ASTER) Global Digital Elevation Model (GDEM v2) data were obtained from the US Geological Survey via the Earthexplorer portal (https://earthexplorer.usgs.gov/), which have an overall accuracy c. 17 m at the 95% confidence interval and were used to derive a 100 m contour layer using the QGIS contour extraction tool. Google satellite imagery was accessed using the QGIS OpenLayers plugin and used to inform digitisation of the watercourses within the study area. All data were projected using the WGS84, EPSG 4326 coordinate reference system.

De novo microsatellite development

A single sample from the Tugela river was sequenced in one flowcell of an Illumina MiSeq following library construction using the Nextera® kit, yielding 2 x 6,465,564 reads of 250 base pair (bp) length. Using the methods of Griffiths et al. [1], microsatellite primers were designed within Galaxy [2,3,4]. Briefly, reads were subject to quality filtering and trimming with Trimmomatic [5] (leading = 3, trailing = 3, sliding window – window size = 4bp, quality = 20; minlen = 50bp), yielding 2 x 3,756,407 reads. Paired reads were then assembled using PANDAseq [6] where possible, and microsatellite loci were searched for only in successfully assembled reads to increase PCR success rates (Fox et al. unpublished). Potentially amplifiable microsatellite loci were identified in the assembled reads using Pal_finder [7], and primers designed using Primer3 [8,9]. Primers for 36 tri- and tetra-nucleotide loci were selected for further testing using Pal_filter [1].

To ensure primers were developed that would amplify across all the potential genetic variation from the region, initial primer testing was conducted on a total of eight individuals from across the sampling range. All primer pairs were first tested in singleplex PCR using the Type-It® Microsatellite PCR kit (Qiagen, Hilden, Germany) in 5µl reactions, with the following cycling conditions: 95°C for 5 minutes, 35 x (95°C for 30 seconds; 60°C for 90 seconds; 72°C for 30

seconds), 60°C for 30 minutes. PCR products were checked on a 1% agarose electrophoresis gel for the presence of one or two bands to show successful amplification. Primer pairs that produced zero or >2 bands were discarded from further testing.

Twenty-seven loci produced satisfactory PCR results, and these amplicons were then labelled with the 6-FAM fluorophore (Thermo-Fisher Scientific, Waltham, USA) using the 3-primer approach developed by Culley et al. [10]. PCR products were checked again on a 1% agarose gel to confirm consistent amplification, then sent for fragment length analysis on an ABI 3730 DNA Analyzer with the GeneScan™ LIZ1200 size standard (both Thermo-Fisher Scientific, Waltham, USA) at the University of Manchester DNA Sequencing Facility.

Alleles were scored in Genemapper v.3.7 (Thermo-Fisher Scientific, Waltham USA) and binned using MsatAllele v1.02 [11]. Loci that were difficult to score, produced more than two alleles per locus or did not amplify consistently were discarded from further analysis. The remaining 17 loci were combined in five multiplex reactions using three universal tail and fluorophore combinations [10,12] (see Table S1 for primer combinations in each multiplex). After optimization, PCR conditions for the multiplexes were modified from the above as follows: For multiplexes 1, 2 and 3, the annealing temperature was changed to 66°C; for multiplex 5 the cycle number was decreased to 32; and for multiplex 4 the annealing temperature was changed to 62°C and the cycle number to 30. Six primers were removed from the final analyses due to significant linkage disequilibrium.

Table S1: Locus and primer characteristics for *de novo Amietia hymenopus* microsatellites

MP: Multiplex; T: Universal tail and fluorophore combination (B = 6FAM-GCCTCCCTCGCGCCA [12], G = HEX-CGGAGAGCCGAGAGGTG [12], R = PET-CACTGCTTAGAGCGATGC [10]); Na: number of alleles per locus; N: average null allele frequency.

Locus	Motif	Primer sequence (5'→ 3')	MP	T	Na	Size range (bp)	N	GenBank accession no.
AHym8	ATT	F: TCTGGGACTGGGATTGACCTAGTGG<br>R: GCAGTAAAGCAGAGCAGCCAATTCC	4	R	7	380-397	0.000	KX772181
AHym12	AATC	F: ACACATGGCATTTTAACATGTTGGG<br>R: TAAATGAAATGTGATGGCGTGTCGG	3	R	7	335-368	0.029	KX772185
AHym7	ATT	F: GCTTGATGTACATTAGCGTGATCAATAGG<br>R: CGGATCCTTCTGAGGTTCTAAGGGG	1	B	5	258-292	0.038	KX772176
AHym25	ATCT	F: AGGTGTCCCTCTTTTCCCTCTCAGG<br>R: TGCACCAACCCATTTATATATCCTGACC	1	R	27	440-569	0.008	KX772179
AHym19	ATCT	F: TATTTCGGGGACCAGGGGATGC<br>R: CCTGAACTGACCAAGTGTCTGGACC	2	G	24	373-470	0.094	KX772177
AHym22	ATT	F: GCTGATGTACCGATTGTCTGCATCC<br>R: GGATACTTAGCTTAACAAAACCATGCAGC	2	B	7	256-274	0.005	KX772178
AHym26	TCTG	F: ATCTCACGGTCCCTCAGACTGC<br>R: CAGTGCCAATAAGACATGCCAGTCC	2	B	17	626-691	0.022	KX772180

AHym11	AAAG	F: GGCAGGAGTGAGAGAAAGAAAGG	5	B	28	533-619	0.180	KX772184
		R: AGCATGCTCTACATACAGCACCACC						
AHym23	AAAG	F: ATAAATACACCCCAATGCCCTGTCC	5	G	23	362-504	0.139	KX772183
		R: AGCAATGCTTTTGGAGAGGTAGAGC						
AHym24	TCTG	F: AGGAATGGTGTCTGGAAAACAGGG	5	B	7	300-340	0.022	KX772182
		R: GAGTGCTGATAGTCTGCCTGCC						
AHym27	ATCT	F: TGAGGGTACCTGCTCTCGTATTGGG	5	R	6	382-406	0.089	KX772186
		R: TCAGAAAGCCTACAGGCAGTGATGG						

Detailed Methods for Population Genetics Analyses

Quality control and summary statistics

We tested for linkage disequilibrium amongst loci in Genepop on the Web v4.2 [13,14], and corrected significance following Benjamini and Yekutieli [15] and Narum [16]. We calculated observed heterozygosity, expected heterozygosity, the inbreeding coefficient and probability of deviation from Hardy-Weinberg equilibrium (HWE) ($5x10^4$ permutations) in GenoDive v2.0b23 [17]. We assessed null allele frequency following the expectation maximization (EM) algorithm using FreeNA [18,19].

Structure

We used loci that did not deviate from HWE, the admixture and correlated allele frequency models, without sampling locations as priors, with $1x10^5$ burn-in and $2x10^5$ Monte Marco Markov Chain repetitions. We tested *K* from one to eight with 20 iterations.

Inest

When testing for both heterozygosity excesses and M-Ratio deficiencies, we used the two-phase mutation model, and tested significance using the Wilcoxon signed-rank test with 1×10^6 permutations.

Geneclass

We used GENECLASS2 v2.0 (Piry *et al.,* 2004) to identify putative first generation migrants using the L_{home} likelihood computation and the Bayesian likelihood criteria of Rannala and Mountain [20] with Monte Carlo resampling probability computation [21] ($p < 0.01$).

Summary Statistics for Population Genetics Analyses

Between 9 and 27 individuals per site were successfully genotyped. No significant linkage disequilibrium was found amongst loci. Per population per locus null allele frequency ranged from zero (in multiple population and loci combinations) to 0.315 (Khubnam, AHym 11), and averaged zero (AHym 8) to 0.180 (AHym 11) across all sites (Table S1); however global F_{ST} only declined very slightly when ENA correction for null alleles was applied (0.122 with correction as opposed to 0.124 without correction) and therefore all loci were retained for further analyses. Number of alleles per locus ranged from 5 (AHym 7) to 28 (AHym 11). Observed heterozygosity over all loci ranged from 0.418 (Ribbon Falls) to 0.745 (Nampolice) (Table S3). Three loci (AHym 19, AHym 23 and AHym 11) significantly deviated from HWE overall (but did not deviate in all sites when considered separately; Table S3).

Table S2

Population pairwise F_{ST} values with ENA correction for null alleles (below diagonal) and Jost's *D* (above diagonal).

	Khubnam	Vemvane	Nampolice	Tugela	Tugela 2	Tukelahed	Bilanjil	Ribbon Falls
**Khubnam**	-	0.239	0.319	0.215	0.211	0.262	0.186	0.502
**Vemvane**	0.065	-	0.11	0.213	0.139	0.247	0.224	0.464
**Nampolice**	0.086	0.045	-	0.318	0.238	0.37	0.38	0.561
**Tugela**	0.053	0.068	0.104	-	0.014	0.066	0.118	0.512
**Tugela 2**	0.054	0.045	0.083	0.008	-	0.087	0.148	0.534
**Tukelahed**	0.081	0.089	0.132	0.024	0.032	-	0.158	0.494
**Bilanjil**	0.039	0.072	0.124	0.035	0.043	0.053	-	0.429
**Ribbon Falls**	0.263	0.242	0.292	0.246	0.263	0.258	0.227	-

Table S3

Descriptive statistics for eleven *Ameitia hymenopus* microsatellite loci across eight sampling sites

n: number of samples successfully genotyped; H_O: observed heterozygosity; H_E: expected heterozygosity; AR: rarefied allelic richness (Khubnam excluded due to low ample size), F_{IS}: inbreeding coefficient with significant values highlighted in bold (populations overall out of HWE across all loci are with F_{IS} values denoted by *).

Site name	n	H_O	H_E	F_{IS}	AR
Khubnam	9	0.515	0.753	0.316	-
Vemvane	27	0.605	0.714	0.153	5.74
Nampolice	19	0.745	0.696	-0.070*	4.41
Tugela	20	0.659	0.768	0.142	7.27
Tugela 2	18	0.654	0.761	0.140	6.55
Tukelahed	18	0.706	0.719	0.018*	5.55
Bilanjil	14	0.591	0.772	0.235	6.59
Ribbon Falls	23	0.418	0.436	0.041*	2.88

Table S4

Analysis of Molecular Variance (AMOVA) within and among *A. hymenopus* individuals, sampling sites and two possible population group scenarios (two groups: Ribbon Falls in one group, all other sites in another; three groups: As previously, but Vemvane and Nampolice in new group).

Scenario	Nested analysis	SSD	Variance-components	% variation	F-statistcs
**Two groups**	Within individuals	510.50	3.449	71.8%	0.282
	Among individuals, within sites	593.11	0.394	8.2%	0.102
	Among sites, within groups	81.83	0.267	5.6%	0.065
	Among groups	70.124	0.694	14.4%	0.144
**Three groups**	Within individuals	510.50	3.449	77.3%	0.227
	Among individuals, within sites	593.11	0.394	8.8%	0.102
	Among sites, within groups	48.50	0.162	3.6%	0.040
	Among groups	103.46	0.460	10.3%	0.103

References

1. Griffiths SM, Fox G, Briggs PJ, Donaldson IJ, Hood S, Richardson P, et al. A Galaxy-based bioinformatics pipeline for optimised, streamlined microsatellite development from Illumina next-generation sequencing data. Conserv Genet Resour. 2016;8:481–86.

2. Giardine B, Riemer C, Hardison RC, Burhans R, Elnitski L, Shah P, et al. Galaxy: a platform for interactive large-scale genome analysis. Genome Res. 2005;15:1451-1455.

3. Blankenberg D, Von Kuster G, Coraor N, Ananda G, Lazarus R, Mangan M, et al. Galaxy: a web-based genome analysis tool for experimentalists. Curr Protoc Mol Biol. 2010;89:19.10:19.10.1-19.10.21.

4. Goecks J, Nekrutenko A, Taylor J, The Galaxy Team. Galaxy: a comprehensive approach for supporting accessible, reproducible and transparent computational research in the life sciences. Genome Biol. 2010;R26.

5. Bolger AM, Lohse M, Usadel B. Trimmomatic: a flexible trimmer for Illumina sequence data. Bioinformatics. 2014;30:2114-20.

6. Masella AP, Bartram AK, Truszkowski JM, Brown DG, Neufeld JD. PANDAseq: paired-end assembler for Illumina sequences. BMC Bioinformatics, 2012;13:31.

7. Castoe T, Poole A, de Koning A, Jones KL, Tomback DF, Oyler-McCance SJ, et al. Rapid microsatellite identification from Illumina paired-end genomic sequencing in two birds and a snake. PLoS ONE. 2012;7:e30953.

8. Untergasser A, Cutcutache I, Koressaar T, Ye J, Faircloth BC, Remm M, et al. Primer3--new capabilities and interfaces. Nucleic Acid Res. 2012;40:115.

9. Koressaar T, Remm M. Enhancements and modifications of primer design program Primer3. Bioinformatics. 2007;23:1289–91.

10. Culley TM, Stamper TI, Stokes RL, Brzyski JR, Hardiman NA, Klooster MR, Merritt BJ. An efficient technique for primer development and application that integrates fluorescent labeling and multiplex PCR. Applications in Plant Sciences. 2013;1:1300027.

11. Alberto F. MsatAllele_1.0: an R package to visualize the binning of microsatellite alleles. J Hered. 2009;100:394–7.

12. Blacket M., Robin C., Good R., Lee S., Miller A. Universal primers for fluorescent labelling of PCR fragments- an efficient and cost-effective approach to genotyping by fluorescence. Mol Ecol Res. 2012;12:456–63.

13. Raymond M, Rousset F. GENEPOP (Version 1.2): population genetics software for exact tests and ecumenicism. J Hered. 1995;86:248–9.

14. Rousset F. genepop'007: a complete re-implementation of the genepop software for Windows and Linux. Mol Ecol Res. 2008;8:103–6

15. Benjamini Y, Yekutieli D. The control of the false discovery rate in multiple testing under dependency. Ann Stat. 2001;29:1165–88.

16. Narum SR. Beyond Bonferroni: less conservative analyses for conservation genetics. Conserv Genet. 2006;7:783–7.

17. Meirmans PG, Van Tiendener PH. Genotype and Genodive: two programs for the analysis of genetic diversity of asexual organisms. Mol Ecol Notes. 2004;4:792–4.

18.Dempster AP, Laird NM, Rubin DB. Maximum likelihood from incomplete data via the EM algorithm. J R Stat Soc Ser B. 1977;39:1–38.

19. Chapuis MP, Estoup A. Microsatellite null alleles and estimation of population differentiation. Mol Biol Evol. 2007;24:621–31.

20. Rannala B, Mountain JL. Detecting immigration by using multilocus genotypes. Proc Natl Acad Sci USA. 1997;94:9197–201.

21. Paetkau D, Slade R, Burden M, Estoup A. Genetic assignment methods for the direct, real-time estimation of migration rate: a simulation-based exploration of accuracy and power. Mol Ecol. 2004;13:55–65.
